# Brain Lesion

**Published:** 1876-09

**Authors:** 


Brain Lesion—Remarkable Case.—The St. Louis *Clinical Record* publishes the following: A man living near Leavenworth, Kansas, died from morphia a short time since. Previous to his death, during a residence in the penitentiary, he had been in the habit of running wires and nails through his own skull. At post-mortem examination two openings were found in the skull, one made by the prisoner with a brad-awl, near the inferior posterior angle of the right parietal, and another near the superior posterior angle of the same bone. In the brain a wire was found introduced through the upper opening, just missing the superior longitudinal sinus. It had pierced to the base of the brain, a little in front of the fissure of sylvius. The corpus striatum was not wounded. A nail, one and three-fourths inches in length, was found lying beside the wire. Although wires had been removed during life from the lower aperture, no trace of their course was observed. The patient, though of a suicidal tendency, did not appear insane, and could do his work with correctness and understanding.—*Physician and Pharmacist.*

